# Health economic evaluation of acupuncture along meridians for treating migraine in China: results from a randomized controlled trial

**DOI:** 10.1186/1472-6882-12-75

**Published:** 2012-06-14

**Authors:** Zhu-qing Deng, Hui Zheng, Ling Zhao, Si-yuan Zhou, Ying Li, Fan-rong Liang

**Affiliations:** 1 Acupuncture and Tuina College, Chengdu University of Traditional Chinese Medicine, Chengdu, Sichuan, 610075, China

**Keywords:** Acupuncture, Migraine, Health economic evaluation, Shaoyang meridian

## Abstract

**Background:**

To evaluate different types of acupuncture treatment for migraine in China from the perspective of health economics, particularly the comparison between treatment of specific acupoints in Shaoyang meridians and penetrating sham acupoints treatment.

**Methods:**

Data were obtained from a multicenter, randomized controlled trial of acupuncture treatment in patients with migraine. Four-hundred eighty migraineurs were randomly assigned to 3 arms of treatment with genuine acupoints and 1 arm of penetrating sham acupoints. The primary outcome measurement was the cost-effectiveness ratio (C/E), expressed as cost per 1 day reduction of headache days from baseline to week 16. Cost-comparison analyses, differences in the migraine-specific quality of life questionnaire (MSQ), and the incremental cost-effectiveness ratio were taken as secondary outcome measurements. In addition, a sensitivity analysis was conducted.

**Results:**

The total cost per patient was ¥1273.2 (95% CI 1171.3-1375.1) in the Shaoyang specific group, ¥1427.7 (95% CI 1311.8-1543.6) in the Shaoyang non-specific group, ¥1490.8 (95% CI 1327.1-1654.6) in the Yangming specific group, and ¥1470.1 (95% CI 1358.8-1581.3) in the sham acupuncture group. The reduced days with migraine were 3.972 ± 2.7, 3.555 ± 2.8, 3.793 ± 3.6, and 2.155 ± 3.7 in these 4 groups (P < 0.05 for each genuine acupoints group vs the sham group), respectively, at week 16. The C/Es of the 4 groups were 320.5, 401.6, 393.1, and 682.2, respectively. Results of the sensitivity analysis were consistent with that of the cost-effectiveness analysis. The Shaoyang specific group significantly improved in all 3 MSQ domains compared with the sham acupuncture group.

**Conclusions:**

Treatment of specific acupoints in Shaoyang meridians is more cost-effective than that of non-acupoints, representing a dramatic improvement in the quality of life of people with migraine and a significant reduction in cost. Compared with the other 3 groups, Shaoyang-specific acupuncture is a relatively cost-effective treatment for migraine prophylaxis in China.

**Trial registration:**

Clinical Trials.gov NCT00599586

## Background

Migraine is characterized by attacks of moderate to severe pulsating and mostly unilateral headache, lasting from 4 to 72 hours, with associated symptoms of nausea, vomiting, and/or photophobia or phonophobia [1]. According to the results of a number of studies, a 1-year prevalence rate varied from 12% to 28%, with females having higher rates than males [2,3]. Migraine mostly impacts individuals aged 25 to 55 years, the years of strong productivity, thus resulting in a loss of productivity and posing a tremendous economic burden to society [4]. A US study in 1999 showed that the monetary loss associated with lost productivity due to headache attacks was estimated to be in the range of $US64-150 million dollars [5].

Acupuncture, a form of traditional Chinese medicine (TCM) therapy, has been gradually accepted in the West as a complementary therapy for migraine, as in part it has the advantage of having fewer side effects than drugs. Acupuncture was reported as a cost-effective treatment for patients with primary headache, improving health-related quality of life [6,7]. However, whether genuine acupuncture in China is more cost-effective than penetrating sham acupuncture is still unknown.

Therefore, we performed a cost-effectiveness analysis based on data from a randomized controlled trial to investigate the cost-effectiveness of different acupuncture treatments, particularly that of specific acupoints in Shaoyang meridians compared with penetrating sham acupoints.

## Methods

### Study design

This study was a health economic evaluation embedded in a multicenter, randomized controlled trial, which was part of a clinical research project supported by China’s National Key Basic Research Program. The trial protocol and the type of informed consent form were followed the principles of the Declaration of Helsinki and provided and approved by local institutional review boards (including the Ethics Committee of first affiliated Hospital of Chengdu University of TCM, the Ethics Committee of First affiliated Hospital of Hunan University of TCM, the Ethics Committee of TCM Hospital of Wuhan City, the Ethics Committee of teaching Hospital of Ningxia Medicine University). All patients gave written informed consent.

Participants aged 18–65 years who met the diagnosis of migraine for ≥ 1 year with at least 2 migraine attacks per month during the last 3 months before randomization were eligible. This study was carried out from April 2008 to December 2009 in China. All participating acupuncturists were required to be qualified with special training for this trial.

Participants were randomly assigned by a central randomization system into 4 treatment groups using: specific acupoints in Shaoyang meridians (group A), non-specific acupoints in Shaoyang meridians (group B), specific acupoints in Yangming meridians (group C), and non-acupoints in all the limbs (group D). Participants received 20 acupuncture sessions with disposable acupuncture needles over a period of 4 weeks. Outcome measurements were assessed at week 0, 4, 8, and 16. Each follow-up carefully assessed the average state of the 4 weeks prior to any given time point. Further details about the design and main clinical outcomes of this study have been published elsewhere [8,9].

### Cost components

Because this study was carried out in 4 cities Chengdu, Changsha, Wuhan, and Yinchuan in China. We measured costs in Chinese Yuan (CNY, ¥, 1 USD = 6.82 CNY on 31th, December, 2009) [10-13]. Costs were categorized as direct and indirect costs. Direct costs comprised acupuncture fees, drug costs, and examination charges. The standard costs of acupuncture treatment per session were 36, 40, 40, 20 (CNY, ¥) in Chengdu, Changsha, Wuhan, and Yinchuan, respectively. Drug costs included all acute and prophylactic medications taken by participants during the treatment and 3-month follow-up. Examination charges involved routine tests for blood, urine and stool, liver and kidney function, an electrocardiograph at baseline, and a transcranial Doppler ultrasound (TCD) examination at baseline and week 4. Not all participants received all the above tests. For example, some migraineurs refused to have a routine stool test, or some missed the second TCD examination at week 4. We therefore calculated examination charges separately. Indirect costs, which from a societal view arose owing to work incapacity for migraine episodes during the study, were evaluated in terms of days of sick-leave from work or other activities like housework. Indirect costs were more difficult to estimate accurately because we did not have access to individuals' salary information. We roughly estimated average daily wages using data from statistical yearbooks based on participants’ occupations and locations. Transportation costs that participants incurred to receive acupuncture treatments were not taken into account as they were not recorded. As the trial period lasted only 16 weeks, discounting rates were not required.

### Primary outcome measurement

The main outcome was the cost-effectiveness ratio (C/E), expressed as the cost per 1 day reduction of headache days. We evaluated effectiveness in terms of differences in migraine days between baseline and week 16 after randomization. This provided a more informative and less subjective approach than use of other outcomes such as migraine intensity [14].

### Secondary outcome measurements

Secondary outcome measurements included costs, scores on the migraine-specific quality of life questionnaire (MSQ V2.1), and the incremental cost-effectiveness ratio (ICER). In addition, a sensitivity analysis was also performed.

MSQ data were acquired at baseline, 4, 8, and 16 weeks by using the Last Observation Carried Forward (LOCF) method. The endpoint was defined as the last available post-baseline observation. The 14-item MSQ assessed the degree of migraineurs’ quality of life over the past 4 weeks in 3 domains: Role Restrictive (RR), Role Preventive (RP), and Emotional Function (EF) [15]. All 3 MSQ dimensions are scored from 0 to 100, with higher scores indicating better states [16]. The reliability and validity of the MSQ has been demonstrated in numerous studies [17].

### Statistical analysis

For sociodemographic baseline characteristics, analysis of variance was used for continuous variables and the chi-squared test for categorical variables. Moreover, analysis of covariance was used for the primary outcome measurement adjusted for center and baseline values. As cost data were not normally distributed, we used the Kruskal-Wallis test. Two-sided tests were applied for all data, and a P value < 0.05 was considered statistically significant. All analyses were done with SAS statistical software (version 9.2, SAS Institute Inc., Cary, NC, USA) and SPSS statistical software (version 13.0, SPSS Inc., Chicago, IL, USA).

## Results and discussion

### Baseline characteristics

A total of 480 participants enrolled, but 41 dropped out of the study for the following reasons: 3 because of false inclusion, 1 because of data loss of the primary outcome, 15 because of unsatisfactory treatments (group A, 3; group B, 4; group C, 4; group D, 4), 17 because of no specific reason (group A, 7; group B, 5; group C, 3; group D, 2), and 5 because of other reasons, such as work issues (group A, 3; group B, 0; group C, 0; group D, 2). In all, 439 participants received at least 12 acupuncture treatment sessions and 3 months’ follow-up, which constituted the cost-effectiveness analysis sample. Groups were comparable in most baseline characteristics with the exception of days of lost work owing to migraine attacks in the last 4 weeks before randomization. Thus, a difference was also found in the costs of sick days due to migraine attacks before randomization (Table 1).

**Table 1:** Baseline characteristics of migraine patients (ITT)

**Parameters**	**Shaoyang specific group (n = 121)**	**Shaoyang non-specific group (n = 119)**	**Yangming specific group (n = 118)**	**Sham a cupuncture group (n = 118)**	P values*
**Age**	37.1 (11.7)	36.2 (12.4)	36.8 (13.0)	37.5 (12.1)	0.870
**Women**	100 (82.6%)	99 (83.2%)	92 (78.0%)	103 (87.3%)	0.306
**Migraine type**					0.635
**With aura**	18 (14.9%)	14 (11.8%)	12 (10.2%)	12 (10.2%)	
**Without aura**	103 (85.1%)	105 (88.2%)	106 (89.8%)	106 (89.8%)	
**Duration of illness (months)**	119.8 (115.3)	91.8 (78.6)	104.0 (100.7)	102.0 (93.4)	0.172
Migraine days last month**	6.3 (5.1)	5.6 (3.2)	6.1 (4.6)	5.5 (4.0)	0.387
**number of lost work days due to**	3.0(3.2)	3.9(3.0)	3.9 (3.7)	2.9(2.7)	0.014*
migraine attacks last month**					

ITT = Intention-to-treat, defined as the number of patients who received at least one acupuncture treatment session. Data are number (%) or mean (SD).SD, Standard Deviation; * Significant difference, P < 0.05. **The average state of 4 weeks before treatment.

### Cost analysis

Table 2 shows that the wages per lost workday were comparable among the study groups. We found no significant intergroup differences among all items in the direct costs category (P > 0.05). However, we did find significant differences for indirect costs (P < 0.05). There was no difference for charge by week 4; however, we found a statistically significant difference for charge by weeks 8 and 16. The Shaoyang specific group had significantly lower indirect and overall costs compared with the other 3 groups (Table 2).

**Table 2:** Cost-comparison analyses in ¥

**Cost components**	**Shaoyang specific group (n = 108) mean[95% CI]**	**Shaoyang non-specific group (n = 110) mean [95% CI]**	**Yangming specific group (n = 111) mean [95% CI]**	**Sham acupuncture group (n = 110) mean [95% CI]**	P values*
**Wage per lost day**	78.9[70.7;86.9]	78.3[69.2;87.3]	76.0[67.6;84.4]	81.7[73.5;89.8]	0.673
**Costs of low**	257.9[184.5;331.4]	320.4[248.5;392.2]	316.7[243.2;390.2]	238.0[187.5;288.6]	0.044‡*
**productivity before**					
**randomization**					
**Direct costs**					
**Examinations**	349.3[338.7;359.8]	344.7[336.7;352.6]	337.1[326.2;348.0]	343.4[332.7;354.0]	0.358
**Acupuncture**	709.0[659.6;758.5]	685.7[647.5;723.9]	721.0[678.2;763.8]	690.1[642.6;737.5]	1.00
**Drug fees**	2.6[1.27;3.85]	2.7[1.39;3.93]	3.7[0.64;6.78]	5.7[1.54;9.85]	0.578
**Total cost**	1060.9[1005.4;1116.3]	1033.0[990.7;1075.3]	1061.8[1012.3;1111.3]	1039.1[985.7;1092.6]	0.717
**Indirect costs**					
**During treatment**	109.3[68.8;149.7]	188.0 [123.9;252.2]	183.3[122.0;244.9]	153.8[115.7;191.9]	0.005‡*
**Weeks 5-8**	57.6[31.01;84.17]	129.0[87.71;170.2]	130.6[79.2;182.1]	128.6[90.22;167.0]	<0.001‡*
**Weeks 13-16**	45.5[24.0;67.0]	77.7[56.0;99.4]	115.1 [60.3;167.0]	148.6[103,5;193.6]	<0.001‡*
**Total cost**	212.3[132.3;292.3]	394.7[289.3;500.1]	429.0[272.6;585.5]	430.9[327.7;534.1]	<0.001‡*
**Charge by week 4**	1170.1[1097.6;1242.6]	1221.1[1142.5;1299.8]	1245.1[1167.4;1322.8]	1192.9[1128.4;1257.3]	0.231
**Charge by week 8**	1227.7[1137.9;1317.5]	1350.0[1242.1;1457.9]	1375.7[1257.6;1493.8]	1321.5[1240.9;1402.o]	0.029‡*
**Overall cost**	1273.2[1171.3;1375.1]	1427.7[1311.8;1543.6]	1490.8[1327.1;1654.6]	1470.1[1358.8;1581.3]	0.004‡*

‡P values based on the Kruskal–Wallis test;* Significant difference, P < 0.05.

### Quality of life

**Figure 1:**
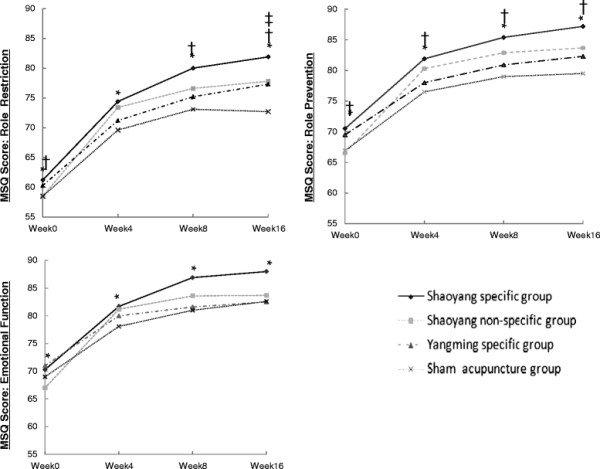
** MSQ mean scores at various time points.** * Shaoyang-specific group vs. sham acupuncture group, P < 0.05. † Shaoyang-non-specific group vs. sham acupuncture group, P < 0.05. ‡ Yangming-specific group vs. sham acupuncture group, P < 0.05.

From a health economic perspective, costs as well as quality of life should be taken into account. Therefore, we used MSQ scores to evaluate quality of life. As 37 participants dropped out during treatment and follow-up (group A: 13, group B: 9, group C: 7, group D: 8), the LOCF method was used for this missing information. Detailed MSQ data can be found in our main clinical publication [9]. Figure 1 shows mean scores at baseline and weeks 4, 8, and 16 for the RR, RP, and EF domains. After acupuncture treatment for all 4 groups, scores in all 3 MSQ domains increased and reached their peaks by the endpoint, except for the RR domain in the sham acupuncture group, whose peak occurred at week 8. Significant differences (P < 0.05) were found in all 3 domains at weeks 4, 8, and 16 for the Shaoyang specific group compared with the sham acupuncture group.

### Cost-effectiveness analysis

The number of reduced days with migraine was 3.972 ± 2.7, 3.555 ± 2.8, 3.793 ± 3.6, and 2.155 ± 3.7 for the Shaoyang-specific group, Shaoyang non-specific group, Yangming specific group, and sham acupuncture group, respectively, at week 16 (P < 0.05 for each of the genuine acupoints group vs the non-acupoints group). Groups using treatment with genuine acupoints resulted in significant improvement compared with the sham acupuncture group, with the best improvement being in the Shaoyang specific group. Based on the cost data and the changes of migraine days from baseline, we performed a cost-effectiveness analysis. The Shaoyang-specific group had a relatively lower C/E than that of the other 3 groups at week 16, and the sham acupuncture group had the highest. Therefore, the negative ICER for treatments in other meridians and non-acupoints indicated that those treatments have a higher expected cost than treatment using Shaoyang specific acupoints (Table 3).

**Table 3:** Cost-effectiveness analysis

**Week 16**	**C (Total cost ¥)**	**E (reduction of migraine days)**	**C/E**	**ΔC/ΔE**
**Shaoyang specific group**				
**Shaoyang specific group**	1273.18	3.972	320.5	-
**Shaoyang non-specific group**	1427.70	3.555	401.6	(370.6)^a^
**Yangming specific group**	1490.84	3.793	393.1	(1216.0) ^a^
**Sham acupuncture group**	1470.06	2.155	682.2	(108.4) ^a^

^a^ Numbers in parentheses are negative ICER values.

### Sensitivity analyses

**Figure 2:**
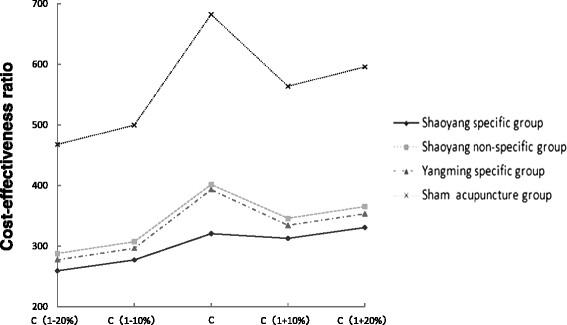
** Sensitivity analyses.** The cost-effectiveness ratio varied by acupuncture fees, which were allowed to drift up and down by 10%-20%. C = Total cost (CNY).

Sensitivity analyses are used to test different assumptions and estimate the influence due to different cost changes. In addition to performing a sensitivity analysis, to improve the external validity of our health economic results we varied acupuncture fees within realistic ranges, taking it as the most important study parameter. During the course of implementing acupuncture treatment, supposing the price fluctuates by 10%-20% according to different hospitals, we conducted a sensitivity analysis. As indicated in Figure 2, the 4 lines show the C/E fluctuation corresponding to the changes of acupuncture fees. The Shaoyang specific group was always lower than the other 3 groups as acupuncture fees were allowed to drift up and down by 10%-20%. We could estimate that the results of the cost-effectiveness analysis appeared steady to cost changes.

### Discussion

Among the groups studied, we found that acupuncture using specific acupoints in Shaoyang meridians was the most efficacious and least costly. The sensitivity analysis was consistent with the cost-effectiveness analysis. This result met what we found in the clinical trial whereby acupoints were more efficacious than non-acupoints at managing migraine. The 4 groups showed similar improvements in weeks 5 to 8; however, there was a significant reduction of migraine days during weeks 13–16 [9]. According to TCM theory, genuine acupoints treatment is more effective than non-acupoints treatment based on specific physiological effects related to meridians and collections of meridian Qi. The findings of this study, from the perspective of health economics, support the use of Shaoyang specific acupoint treatment for migraine compared with the other acupuncture treatments studied.

MSQ outcomes were better for participants in the Shaoyang specific group compared with the other groups. Based on the evaluation of MSQ scores, the Shaoyang specific group participants showed significant and sustained improvement in daily activities and well-being for up to 3 months.

The difference in indirect costs of low productivity at baseline was probably due to sample size. However, indirect costs measured at the following 3 time points probably were not influenced by the small sample size because penetrating sham acupuncture treatment, having the lowest indirect costs, was superior to the 3 genuine acupuncture groups at the beginning.

The study has the following limitations. First, the study design did not have routine drug therapy as a control group. The clinical trial was designed to investigate the efficacy of acupuncture (verum acupuncture and penetrating sham acupuncture), so usual care was not involved. Second, cost-utility analysis was not possible to use here. The disease-specific MSQ were chosen instead of the 36-item short-form health survey (SF-36), which is a generic scale not necessarily affected by a specific disease. We could not obtain quality-adjusted life years(QALY). The value of our analysis was limited to a single measure, which could not be used to compare situations with different benefits. A follow-up visit at week 12 was not initially included in the study design for collecting data from week 9 to week 12. Therefore, we were not able to estimate costs due to lost productivity and drug fees paid by participants during weeks 9–12.

Acupuncture, with its unique feature and advantages, has been widely applied in the treatment of many neurological disorders; however, its benefits still lack strong evidence from the perspective of health economics. Health economics deals with optimizing the allocation of healthcare resources. Because of the limited health resources in China, more health economics evaluations are needed to provide evidence for optimal allocation of healthcare resources. To our knowledge, this is the first economic evaluation of acupuncture treatment for migraine in China.

## Conclusions

Our study showed that treatment using specific acupoints in the meridians of Shaoyang is more cost-effective than that using non-acupoints, representing a dramatic improvement in the quality of life for migraineurs and a significant reduction in cost. Compared with the other 3 groups, the method of selecting specific acupoints in the meridians of Shaoyang is a relatively cost-effective treatment for migraine prophylaxis.

## Competing interests

The authors declare that they have no competing interests.

## Authors’ contributions

ZQD drafted the manuscript. HZ contributed to the study concept and design. All authors contributed to reading and revising the manuscript and all have approved the final manuscript.

## Pre-publication history

The pre-publication history for this paper can be accessed here:

http://www.biomedcentral.com/1472-6882/12/75/prepub
